# Investigation of *Cryptosporidium* spp. and *Enterocytozoon bieneusi* in free-ranged livestock on the southeastern Qinghai–Xizang Plateau, China

**DOI:** 10.1186/s12879-025-10737-5

**Published:** 2025-03-13

**Authors:** Xiaoxue Peng, Xu Wang, Jinhua Jian, Qingqiu Zuo, Hua Liu, Yaxue Wang, Yaxin Su, Jianping Cao, Bin Jiang, Yujuan Shen

**Affiliations:** 1 National Institute of Parasitic Diseases, Chinese Center for Disease Control and Prevention (Chinese Center for Tropical Diseases Research), National Key Laboratory of Intelligent Tracking and Forecasting for Infectious Diseases, NHC Key Laboratory of Parasite and Vector Biology, WHO Collaborating Centre for Tropical Diseases, National Center for International Research on Tropical Diseases, Shanghai, 200025 China; 2 School of Basic Medical Sciences and Forensic Medicine, Hangzhou Medical College, Hangzhou, Zhejiang 310000 China

**Keywords:** *Cryptosporidium* spp., *Enterocytozoon bieneusi*, Yaks, Tibetan sheep, Horses, Zoonotic, Shiqu

## Abstract

**Background:**

*Cryptosporidium* spp. and *Enterocytozoon bieneusi* are zoonotic pathogens with global distribution, infecting humans and various livestock. For yaks, Tibetan sheep and horses, the traditional grazing models still hold a main position. After these animals become infected, it not only hinders the development of animal husbandry but also increases the risk of *Cryptosporidium* spp. and *E. bieneusi* transmission between livestock and herdsmen.

**Methods:**

In the present study, a total of 750 fecal samples were collected from yaks, Tibetan sheep and horses in Shiqu County, Sichuan Province, from July to August 2023, and were analyzed by nested Polymerase chain reaction (PCR) amplification of the small subunit ribosomal RNA (SSU rRNA) gene of *Cryptosporidium* spp. and internal transcribed spacer (ITS) gene of *E. bieneusi*.

**Results:**

The prevalence of *Cryptosporidium* spp., and *E. bieneusi* were 2.1% (16/750) and 1.5% (11/750), respectively. Mixed infections of *Cryptosporidium* spp. and *E. bieneusi* were detected in two samples. Among these positive fecal samples, one *Cryptosporidium* species (*Cryptosporidium suis*) was identified in the yaks (*n* = 11), Tibetan sheep (*n* = 1), and horses (*n* = 4). Three *E. bieneusi* genotypes, including a known genotype BEB4 and two novel ones SQY1 and SQY2, were identified in the yaks (*n* = 7), while in Tibetan sheep (*n* = 4) only the known genotype BEB4 was detected. The novel genotype SQY1 was grouped into the human-pathogenic Group 1, and the known genotype BEB4 and the novel genotype SQY2 were grouped into Group 2. *Enterocytozoon bieneusi* was not detected in horses.

**Conclusions:**

*Cryptosporidium suis* was identified in yaks and horses while zoonotic *E. bieneusi* genotype BEB4 in Tibetan sheep for the first time, expanding their host ranges. These findings suggested that yaks, Tibetan sheep and horses could act as potential sources of human *Cryptosporidium* spp. and *E. bieneusi* infections, implying that the presence of zoonotic species/genotypes could pose a threat to public health.

**Supplementary Information:**

The online version contains supplementary material available at 10.1186/s12879-025-10737-5.

## Background

*Cryptosporidium* spp. and *Enterocytozoon bieneusi* are important zoonotic intestinal pathogens responsible for parasitic diarrhea diseases worldwide, posing a great threat to public health [1]. Both of them can infect humans and virtually all vertebrate animals [2–5]. These two pathogen infections affect the gastrointestinal system of hosts and mainly bring about diseases characterized by diarrhea. The two parasitic diseases can progress to life-threatening diarrhea in immunocompromised/immunodeficient individuals [6]. Infections occur mainly by fecal-oral transmission after ingestion of infective *Cryptosporidium* spp. oocysts or *E. bieneusi* spores, usually via contaminated water and food or direct contact with infected hosts [7]. *Cryptosporidium* spp. oocysts and *E. bieneusi* spores released by infected hosts show prolonged survival in aquatic environments, a trait contributing to public health risks [8]. Specifically, *Cryptosporidium* spp. is identified as the leading cause of 1,227 reported waterborne outbreaks, while *E. bieneusi* combined with *Encephalitozoon intestinalis* was linked to a 200-case waterborne outbreak in France. These findings demonstrate that the environmental persistence of these protozoan pathogens enhances their outbreak potential [9, 10]. *Enterocytozoon bieneusi*, one of 17 microsporidia known to be infectious to humans, is mainly associated with immunocompromised individuals, causing wasting syndrome [11]. *Cryptosporidium* spp. and *E. bieneusi* lead to cryptosporidiosis and microsporidiosis, respectively, in humans and animals including livestock [12]. Based on economic and health considerations, individuals, especially those who work with livestock should be made aware of the potential zoonotic transmission of cryptosporidiosis and microsporidiosis due to contact with infected animals [13].

Currently, more than 49 species of *Cryptosporidium* with 120 genotypes have been described in a wide variety of hosts, with 23 species and two genotypes reported in humans, including *Cryptosporidium hominis*, *Cryptosporidium parvum*, *Cryptosporidium meleagridis*, *Cryptosporidium canis*, *Cryptosporidium felis*, *Cryptosporidium ubiquitum*, *Cryptosporidium cuniculus*, *Cryptosporidium viatorum*, *Cryptosporidium muris*, *Cryptosporidium andersoni*,* Cryptosporidium erinacei*, *Cryptosporidium tyzzeri*, *Cryptosporidium bovis*, *Cryptosporidium suis*, *Cryptosporidium scrofarum*, *Cryptosporidium occultus*, *Cryptosporidium xiaoi*, *Cryptosporidium fayeri*, *Cryptosporidium ditrichi*, *Cryptosporidium equi* (horse genotype), *Cryptosporidium wrairi*, *Cryptosporidium mortiferum* (chipmunk genotype I), *Cryptosporidium baileyi*, mink genotype, and skunk genotype [13–15]. Among them, *C. hominis* and *C. parvum* are responsible for the majority of human infections [13]. Similarly, based on the molecular characterization of the ITS gene locus of *E. bieneusi*, at least 819 unique genotypes have been identified [16]. Phylogenetic analysis has divided these genotypes into 15 distinct groups (Groups 1–15), with more than 90% of the genotypes belonging to Groups 1 and 2 [17, 18]. The majority of the zoonotic genotypes are clustered in Group 1 [5]. With accumulating molecular epidemiological data of *E. bieneusi*, some genotypes (I, J, BEB4, and BEB6) in Group 2 have also been found in both humans and animals [5]. The other groups (Groups 3–15) are mostly host-adapted with limited zoonotic potential [8, 19]. Some genotypes have only been reported in dogs, including genotypes PtEb IX, CD7, CD8, and WW8 in Group 11 and genotype CD5 in Group 7 [5].

According to the studies reported so far, most *Cryptosporidium* species and genotypes identified in livestock have been found in humans especially *C. parvum* and *C. hominis* [6, 20, 21]. Livestock infected with these protozoa indicates that there is a significant risk of *Cryptosporidium* spp. transmission between livestock and humans. Similarly, previous studies have reported that the genotypes of *E. bieneusi* detected in Tibetan sheep and yaks were also found in humans including genotype PigEBITS5 in Group 1 and genotypes I, J, and BEB4 in Group 2, respectively, suggesting that there is a zoonotic potential [22–24]. To date, there have been more than 40 *E. bieneusi* genotypes identified in equines including some zoonotic genotypes D and Peru8 in Group 1 and genotype BEB6 in Group 2 [5].

To date, epidemiological data regarding *Cryptosporidium* spp. and *E. bieneusi* distribution in yaks, Tibetan sheep, and horses in Shiqu is absent. Therefore, the current study aimed to investigate the infection of *Cryptosporidium* spp. and *E. bieneusi* in livestock on the plateau and their genetic characterization at the species/genotype level.

## Materials and methods

### Study area

Shiqu County (32°58′36.9″ N, 98°6′4.7″ E) is located in the northwest of Sichuan Province on the southeastern edge of the Qinghai–Xizang Plateau and has high-altitude climate characteristics of low temperature, low oxygen, and high ultraviolet. In this area, animal husbandry is the main economic activity, and yaks, Tibetan sheep, and horses are common economic animals [25]. Thus, individuals who come into close contact with these animals for occupational (herders) or recreational reasons (travelers) are at a high risk of intestinal parasitic infections. The role of these animals in zoonotic pathogen infection is gaining more traction in this area.

A molecular epidemiological investigation of *Cryptosporidium* spp. and *E. bieneusi* was carried out in Shiqu County (part of Ganzi Tibetan Autonomous Prefecture of Sichuan Province) at the junction of Sichuan, Qinghai and Xizang provinces on the southeast margin of the Qinghai–Xizang Plateau, China. The county has an average altitude of 4, 200 m, characterized by low temperature, low oxygen, and high ultraviolet. Due to the alpine meadows covering the vast majority of the territory, animal husbandry is the main economic component in the area.

### Sample collection

From July to August 2023, a total of 750 fecal samples (350 from yaks, 250 from Tibetan sheep, and 150 from horses) were randomly collected from pastures in two townships (Sexu and Yiniu) in Shiqu County. All the animals naturally graze on rangeland and share the grazing lands and drinking water. All samples were collected from grazing yaks, Tibetan sheep, and horses with no clinical signs. All the fecal samples were collected from the top layer of feces when grazing livestock defecated on the ground to avoid contamination.

In the selected area, each fecal sample from livestock was collected using a new disposable tool to avoid cross-contamination, with the fresh samples stored in 50 ml centrifuge tubes. Each sample was labeled with the date, location, breed, and unique number and then kept in a freezer (-20℃) of the Shiqu County Center for Disease Control and Prevention. Finally, they were transported in ice packs to our laboratory at -80℃ in Shanghai for further molecular analysis.

### DNA extraction

The fecal samples were diluted with 0.9% normal saline and filtered through a 100-mesh nylon mesh filter to reduce interference of crude fiber and impurities in animal manure. The filtrates were centrifuged at 3, 000 g for 10 min to enrich *Cryptosporidium* spp. oocysts and *E. bieneusi* spores. Before DNA extraction, each fecal sample from 50 ml centrifuge tubes was transferred (up to 200 mg) to a 2 ml microcentrifuge tube with 100 mg of ceramic balls. Genomic DNA was extracted using the QIAamp Fast DNA Stool Mini Kit (cat. #51604; Qiagen, Hilden, Germany) according to the manufacturer’s recommended protocol, with an elevated lysis temperature of 95℃ to guarantee high quality and DNA yield. Extracted DNA was stored at − 80 °C before analysis by PCR.

### PCR amplification

**Fig. 1:**
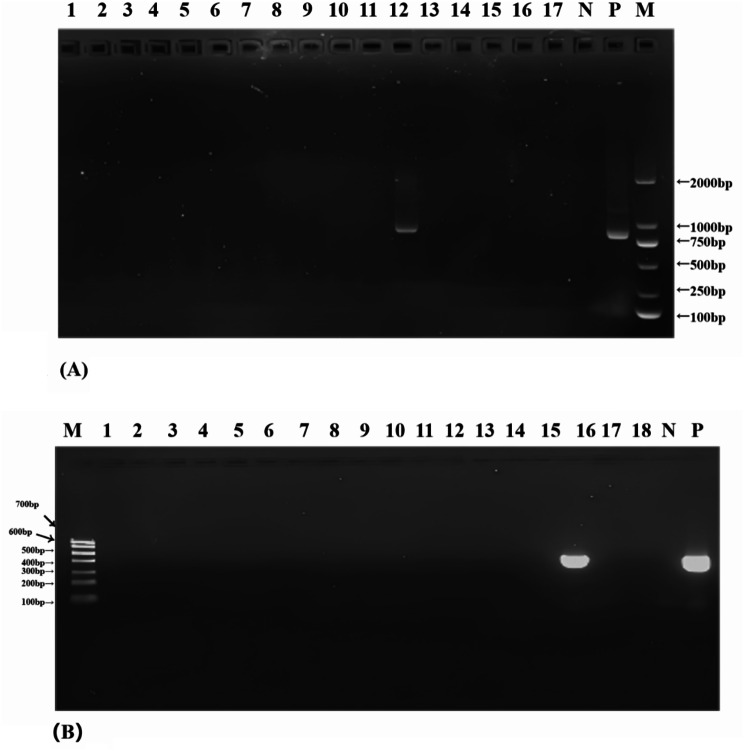
(**A**) Representative agarose gel image showing PCR amplification products of the SSu rRNA gene of *Cryptosporidium* spp. (expected band size 830 bp). Lanes 1-7: Samples; N: negative control; P: Postive control; M: DL 2000 DNA marker. (**B**) Representative agarose gel image showing PCR amplification products of the ITS gene of *Enterocytozoon bieneusi* (expected band size 410 bp) Lanes 1-18: Samples; N: negative control; M: DL 700 DNA marker

*Cryptosporidium* spp. and *E. bieneusi* were screened by nested PCR amplification of a fragment (~ 830 bp) of the SSU rRNA gene and a fragment (~ 410 bp) of the internal transcribed spacer (ITS) region of the rRNA gene as previously described, respectively [26, 27]. The primers and cycling parameters were used in the PCR analysis of the two gene targets as described by Huang et al. (2016) and Mirjalali et al. (2015) (Additional file 1: Table S1). Reaction mixtures were performed using the 2×TransTaq^®^-T PCR SuperMix (+ dye) (TransGen Biotech, Beijing, China), according to the manufacturer’s instruction. The reaction volume was 25 µl containing 12.5 µl of 2×TransTaq^®^-T PCR SuperMix (+ dye), 9.5 µl of nuclease-free water, 1 µl of template DNA and 10µM of each primer. *Cryptosporidium* spp. was screened based on the SSU rRNA gene by nested PCR as the following conditions: 95 ℃ for 5 min; followed by 35 cycles of 95 ℃ for 40s, annealing at 55 ℃ for 45s, and 72 °C for 45s; and a final extension at 72 °C for 10 min. Conditions for the secondary PCR were identical to the primary PCR. *Enterocytozoon bieneusi* was screened based on the ITS region of the rRNA by nested PCR as the following conditions: 95 ℃ for 5 min; followed by 35 cycles of 95 ℃ for 40s, annealing at 53 ℃ for 45s for the first round of PCR or 55℃ for 40s for the second round, and 72 °C for 45s; and a final extension at 72 °C for 10 min. All the samples were analyzed twice, using nuclease-free water as the negative control and a human-derived *C. parvum* DNA and *E. bieneusi* genotype D DNA as the positive controls (GenBank accession numbers: MF074733; LC436471). The secondary PCR products were separated by 1.5% agarose gel electrophoresis containing GelRed (Biotium Inc., Hayward, CA, USA) and visualized on a UV transilluminator. The partial gel images of *Cryptosporidium* spp. and *E. bieneusi* have been placed in Fig. 1.

### Nucleotide sequencing and analyzing

All of the positive PCR products at the two gene loci were sent to a commercial company (BGI, Shanghai, China) for sequencing using the Sanger dideoxy sequencing method and their respective secondary PCR primers by ABI 3730XL DNA Analyzer (Applied Biosystems, Foster City, USA) and Big Dye Terminator v3.1 Cycle Sequencing Kit (Applied Biosystems). The accuracy of the nucleotide sequence was assured by bi-directional sequencing. Further PCR products were sequenced for some DNA preparations of the expected size, from which we obtained the sequences having single nucleotide substitutions, deletions, or insertions compared to those published in GenBank. The sequences obtained in the present study were assembled using ChromasPro 2.1.6 (http://technelysium.com.au/ChromasPro.html) and aligned using ClustalX 2.0.11 (http://clustal.org) with each other and reference sequences downloaded from GenBank (http://www.ncbi.nlm.nih.gov). The BLAST analysis was used to identify *Cryptosporidium* species/genotypes and *E. bieneusi* genotypes.

### Phylogenetic analyses

Analyses of the phylogenetic relationships of the ITS sequences among the *E. bieneusi* genotypes and the SSU rRNA sequences among *Cryptosporidium* spp. were performed based on the neighbor-joining (NJ) method and the Kimura-2-parameter model using the MEGA 11.0.13. To assess the robustness of the clusters, 1, 000 bootstrap replicates were performed. Reference sequences from GenBank were downloaded, and the sequences were labeled with the National Center for Biotechnology Information (NCBI) accession number, the host origin, and the genotypes for *E. bieneusi*, and species, and accession number for *Cryptosporidium* spp.

### Statistical analyses

To determine the differences in prevalence of *Cryptosporidium* spp. and *E. bieneusi* between sampling sites and among three animal species, the chi-square test and Fisher’s exact test were applied to each of the two variables by using Regression Analysis in SPSS 29.0 software with 95% confidence intervals (CI). *P* values < 0.05 were considered as statistical significance.

## Results

### Occurrence of *Cryptosporidium* spp. And *E. bieneusi*

Of the 750 samples analyzed, 16 (2.1%) and 11 (1.5%) were PCR-positive for *Cryptosporidium* spp. and *E. bieneusi*, respectively. The prevalence of *Cryptosporidium* spp. infection varied by sampling sites: 2.7% (12/450) in Sexu and 1.3% (4/300) in Yiniu (Table 1). Yaks had the highest prevalence (3.1%, 11/350) of *Cryptosporidium* spp., followed by horses (2.7%, 4/150) (χ^2^ < 0.001, *p* = 1.000) and Tibetan sheep (0.4%, 1/250) (χ^2^ = 5.598, *p* = 0.018) (Table 2). *Cryptosporidium* spp. was absent in Tibetan sheep in Sexu. There was no significant difference in prevalence of either *Cryptosporidium* spp. or *E. bieneusi* between sampling sites (χ^2^ = 1.533, *p* = 0.216) and among three animal species.

For *E. bieneusi*, the prevalence rates were 1.3% (6/450) in Sexu and 1.7% (5/300) in Yiniu (χ^2^ = 0.004, *p* = 0.951) (Table 1). Yaks had a slightly higher prevalence of *E. bieneusi* (2.0%, 7/350) than Tibetan sheep (1.6%, 4/250). Meanwhile, *E. bieneusi* was absent in Tibetan sheep in Yiniu and all the investigated horses (Table 2). Additionally, two mixed infection cases of both protozoa were observed in yaks, one from Sexu Town, and the other from Yiniu Village (Table 1).

**Table 1:** Prevalence and species/genotypes of *Cryptosporidium* spp. and *Enterocytozoon bieneusi* in sexu and Yiniu

Sample site	Examined no.	*Cryptosporidium* spp.		*Enterocytozoon bieneusi*		*Cryptosporidium* spp. + *Enterocytozoon bieneusi*	
		Prevalence % (95% CI)	Species (no.)	Prevalence % (95% CI)	Genotype (no.)	Prevalence % (95% CI)	Species/genotype (no.)
SX	450	2.7 (1.4 ~ 4.6)	C. *suis* (12)	1.3 (0.5 ~ 2.9)	BEB4 (6)	0.2 (0.0 ~ 1.2)	*C. suis* + BEB4 (1)
YN	300	1.3 (0.4 ~ 3.4)	*C. suis* (4)	1.7 (0.5 ~ 3.8)	BEB4 (2), SQY1 (2), SQY2 (1)	0.3 (0.0 ~ 1.8)	*C. suis* + SQY2 (1)
Total	750	2.1 (1.2 ~ 3.4)	*C. suis* (16)	1.5 (0.7 ~ 2.6)	BEB4 (8), SQY1(2), SQY2 (1)	0.3 (0.1 ~ 1.0)	*C. suis* + BEB4 (1), *C. suis* + SQY2 (1)

SX: Sexu town, YN: Yiniu village

**Table 2:** Prevalence and distribution of *Cryptosporidium* species and *Enterocytozoon bieneusi* genotypes in livestock

Hosts	Sample sites	Examined no.	*Cryptosporidium* spp.		*Enterocytoon bieneusi*	
			Prevalence % (95% CI)	Species (no.)	Prevalence % (95% CI)	Genotype (no.)
Yak	Sexu	200	4.5 (2.1 ~ 8.4)	*C. suis* (9)	1.0 (0.1 ~ 3.6)	BEB4 (2)
	Yiniu	150	1.3 (0.2 ~ 4.7)	*C. suis* (2)	3.3 (1.1 ~ 7.6)	BEB4 (2), SQY1 (2), SQY2 (1)
Subtotal		350	3.1 (1.6 ~ 5.6)	*C. suis* (11)	2.0 (0.8 ~ 4.1)	BEB4 (4), SQY1 (2), SQY2 (1)
Tibetan sheep	Sexu	150	-	-	2.7 (0.7 ~ 6.7)	BEB4 (4)
	Yiniu	100	1.0 (0.0 ~ 5.4)	*C. suis* (1)	-	-
Subtotal		250	0.4 (0.0 ~ 2.2)	*C. suis* (1)	1.6 (0.4 ~ 4.0)	BEB4 (4)
Horse	Sexu	100	3.0 (0.6 ~ 8.5)	*C. suis* (3)	-	-
	Yiniu	50	2.0 (0.1 ~ 10.6)	*C. suis* (1)	-	-
Subtotal		150	2.7 (0.7 ~ 6.7)	*C. suis* (4)	-	-

The dash “-” indicates negative

### Molecular characteristics and distribution of *Cryptosporidium* species

Based on sequence analysis of the SSU rRNA gene, all 16 *Cryptosporidium*-positive samples were *C. suis* including yaks (*n* = 11), Tibetan sheep (*n* = 1), and horses (*n* = 4) from two pastures. The occurrence frequency of *C. suis* (68.8%, 11/16) was the highest in yaks, followed by horses (25.0%, 4/16), and Tibetan sheep (6.3%, 1/16). Compared with the reference pig-derived *C. suis* sequence (GenBank accession No. MT071826), 13 sequences had 100% homology with it, and the remaining three SSU rRNA sequences differed by one base substitution.

### Molecular characteristics and distribution of *E. bieneusi* genotypes

Phylogenetic characterization of the ITS gene region (243 bp) of 11 *E. bieneusi* isolates revealed three ITS genotypes (one known genotype BEB4 and two novel genotypes SQY1, and SQY2). There were 14 polymorphic sites observed among them (Table 3). Genotype BEB4 was the most common. The three genotypes were identified in seven *E. bieneusi*-positive yaks while one genotype BEB4 in four *E. bieneusi*-positive Tibetan sheep.

**Table 3:** Nucleotide variation at 14 polymorphic sites in the ITS region of the rRNA genes of *Enterocytozoon bieneusi* isolates obtained in this study

Genotype	GenBank ID	Nucleotide at position (ITS)													
		18	33	81	91	93	113	117	129	131	133	137	147	158	219
Known															
BEB4	OM101098	A	A	T	T	T	T	G	A	C	G	T	A	A	A
Novel															
SQY1	PQ628072	**G**	**G**	**C**	T	**C**	**C**	**T**	**G**	**G**	**A**	**C**	**G**	**T**	**G**
SQY2	PQ628069	A	A	T	**C**	T	T	G	A	C	G	T	A	A	A

The bold text indicates different base compared to BEB4

### Phylogenetic analyses of *E. bieneusi* genotypes and *Cryptosporidium* species

**Fig. 2:**
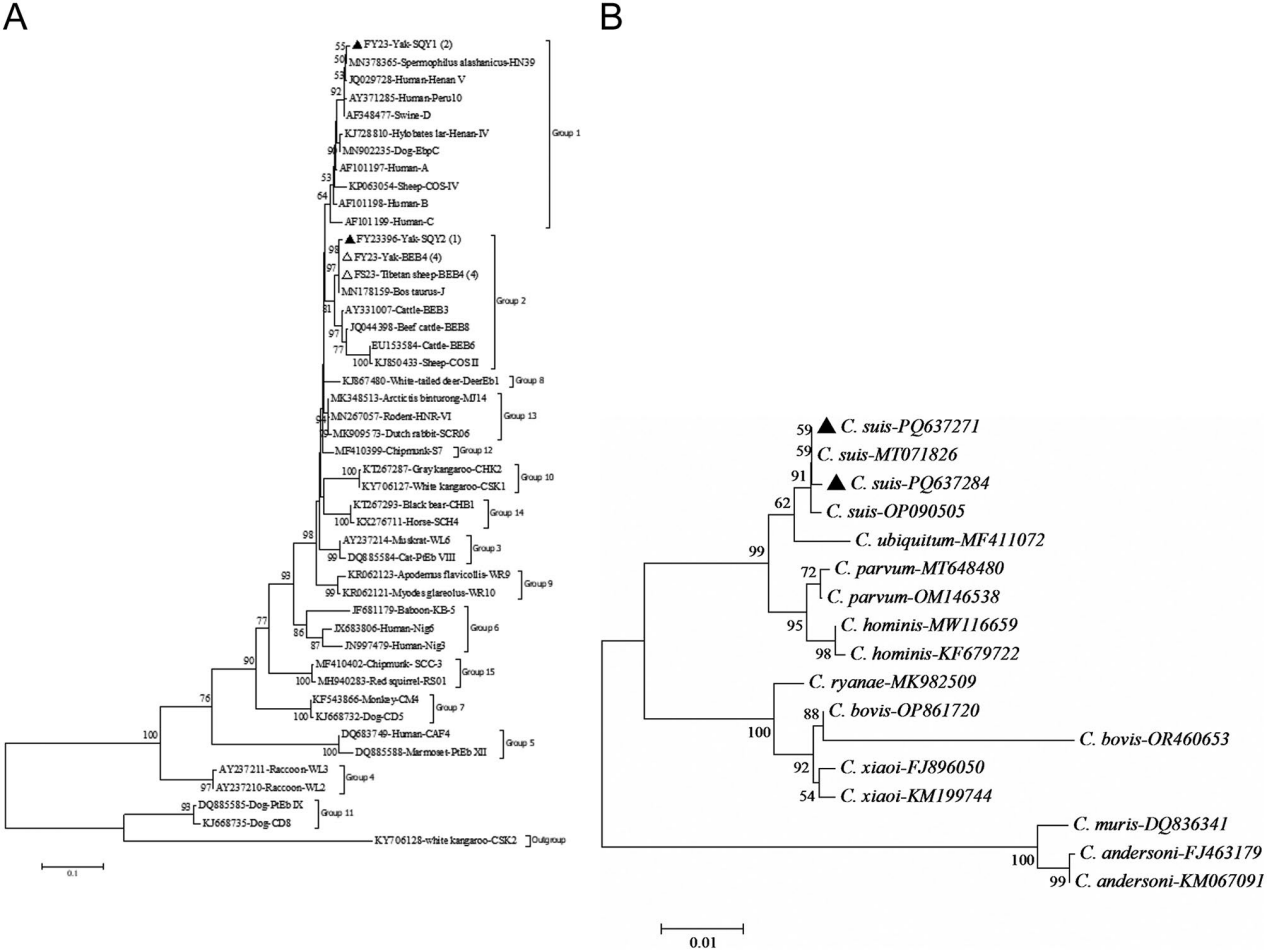
**A** Phylogenetic relationships of *Enterocytozoon bieneusi* genotypes identified in this study and known genotypes deposited in GenBank based on neighbor-joining (NJ) analysis of ITS sequences. Bootstrap values were obtained using 1,000 replicates, with more than > 50% shown on nodes. The sequences are given as accession number, host origin, and genotype designation. The *Enterocytozoon bieneusi* genotype CSK2 (isolated from white kangaroo) was designated as the phylogenetic outgroup. Genotypes marked with open triangles and black triangles are known and novel genotypes identified in this study, respectively. ▲: Novel genotypes from this study. △: Known genotypes from this study. **B** Phylogenetic relationships of *Cryptosporidium* spp. genotypes identified in this study and known genotypes deposited in GenBank based on neighbor-joining (NJ) analysis of SSU rRNA sequences. The *Cryptosporidium suis* cluster (GenBank accession PQ637271) comprises 13 sequences exhibiting 100% sequence identity to reference strain MT071826. The *Cryptosporidium suis* clade (GenBank accession PQ637284) contains three divergent SSU rRNA sequences, each exhibiting a single nucleotide polymorphism relative to reference strain MT071826. ▲: Species from this study

Phylogenetic analysis revealed that one novel genotype fell into zoonotic Group 1, and the others were clustered into Group 2, with increasing zoonotic potential (Fig. 2). The two isolates (genotype SQY1, *n* = 2) belonged to Group 1 from yaks. The other nine sequences clustered into Group 2: yaks (genotypes BEB4, *n* = 4; SQY2, *n* = 1) and Tibetan sheep (genotype BEB4, *n* = 4). *Cryptosporidium suis* was constructed as a phylogenetic tree with the SSU rRNA gene sequences (Fig. 2).

### Nucleotide sequence accession numbers

The detected nucleotide sequences in the present study were deposited in GenBank under accession numbers PQ637271 - PQ637286 for *C. suis*, and PQ628064 - PQ628074 for *E. bieneusi*.

## Discussion

In the present study, the prevalence and genetic characterization of *Cryptosporidium* spp. in free-ranged livestock were analyzed. The occurrence rate of *Cryptosporidium* spp. infection was 2.1% in the investigated animals. Yaks had a slightly higher prevalence of 3.1% than those yaks reported in four studies (0.7–2.5%) in Qinghai [28–31] and in two studies (1.4%, the same prevalence in two studies) in Xizang [32, 33]. However, the present prevalence of *Cryptosporidium* spp. was lower than those in nine studies in Qinghai and Sichuan (11.3–39.7%) [34–43]. In contrast, Tibetan sheep in this study had a similar prevalence to the sheep in Xinjiang (0.9%) [44], which was lower than that in natural grazing Tibetan sheep (15%) in Xizang [23], and the sheep in 11 provinces of China (20.4%) [45]. The prevalence rate in horses (2.7%, 4/150) was coincident with the one reported previously in grazing horses in Xinjiang, China (2.7%, 7/262) [46], which was higher than that in horses in Brazil (0.8%, 3/396) [47], Algeria (2.3%, 5/219) [48], Belgium (2.0%, 8/398) [49], and China (1.8%, 6/333) [50], but lower than that in previous studies in China (36.9%, 161/436) [51], Brazil (19.9%, 39/196; 21.7%, 20/92) [52, 53], and Italy (8.0%, 12/150) [54].

The prevalence of *E. bieneusi* infection was 2.0% (7/350) in yaks and 1.6% (4/250) in Tibetan sheep (Table 2). Moreover, the prevalence of *E. bieneusi* in yaks worldwide can be up to 23.8% [5, 25]. The prevalence of *E. bieneusi* infection in yaks in the present study was distinctly lower than that of yaks in Sichuan (23.8%, 53/223) [25], Qinghai (7.0%, 23/327; 7.2%, 40/554) [55, 56], and the Qinghai–Xizang Plateau (12.9%, 13/101) [30], but higher than one study in Gansu (1.1%, 4/353) [57]. The prevalence of *E. bieneusi* in sheep reported in published studies is high up to 69.3% [58], whereas in goats, it ranges from 7.5 to 32.9% [24]. Compared with the results of the majority of these earlier publications, such as sheep from Brazil (19.2%, 24/125) [59], Sweden (45.0%, 49/109) [60], China (ranging from 4.4 to 69.3% in eight studies) [58, 61–67], and Tibetan sheep from China (9.1%, 10/110; 23.4%, 73/312) [57, 68], the prevalence of *E. bieneusi* infection in Tibetan sheep in this study was relatively lower, but higher than that of blue sheep (1.0%, 1/96) [68]. In addition, the prevalence *E. bieneusi* in horses is up to 30.9% globally [5, 69]. Surprisingly, we did not detect *E. bieneusi* in horses, which has been detected in China (22.5%,75/333; 7.4%, 24/325) [70, 71], in Colombia (10.8%, 21/195) [72], in the Czech Republic (17.5%, 66/377) [73], and in Algeria (6.8%, 15/219) [48]. We hypothesize that it may be related to the low intensity of infection, the immune status of the hosts, and the number of samples. Moreover, the yaks, Tibetan sheep, and horses examined were grazed outdoors on cold, low-oxygen pastures in Shiqu County, conditions which differ from previous reports.

The prevalence of *Cryptosporidium* spp. infection varies in different animals between countries and even between regions of the same country. Yaks, Tibetan sheep, and horses of Shiqu County exhibit considerably low prevalence of *Cryptosporidium* spp. infection due to their environments, since oocysts have a higher survival rate in warm and humid conditions and a lower survival rate in cold, dry environments and harsh climates with high altitudes [20, 30, 74, 75]. One previous study implied that the prevalence of *Cryptosporidium* spp. at an altitude of < 3, 000 m was higher than that at an altitude of > 3, 000 m. Additionally, the temperature was usually high at a low altitude [21]. Furthermore, in the Qinghai–Xizang Plateau, the unique alpine climate limits the survival and dispersal of infective *Cryptosporidium* spp. oocysts [76].

**Fig. 3:**
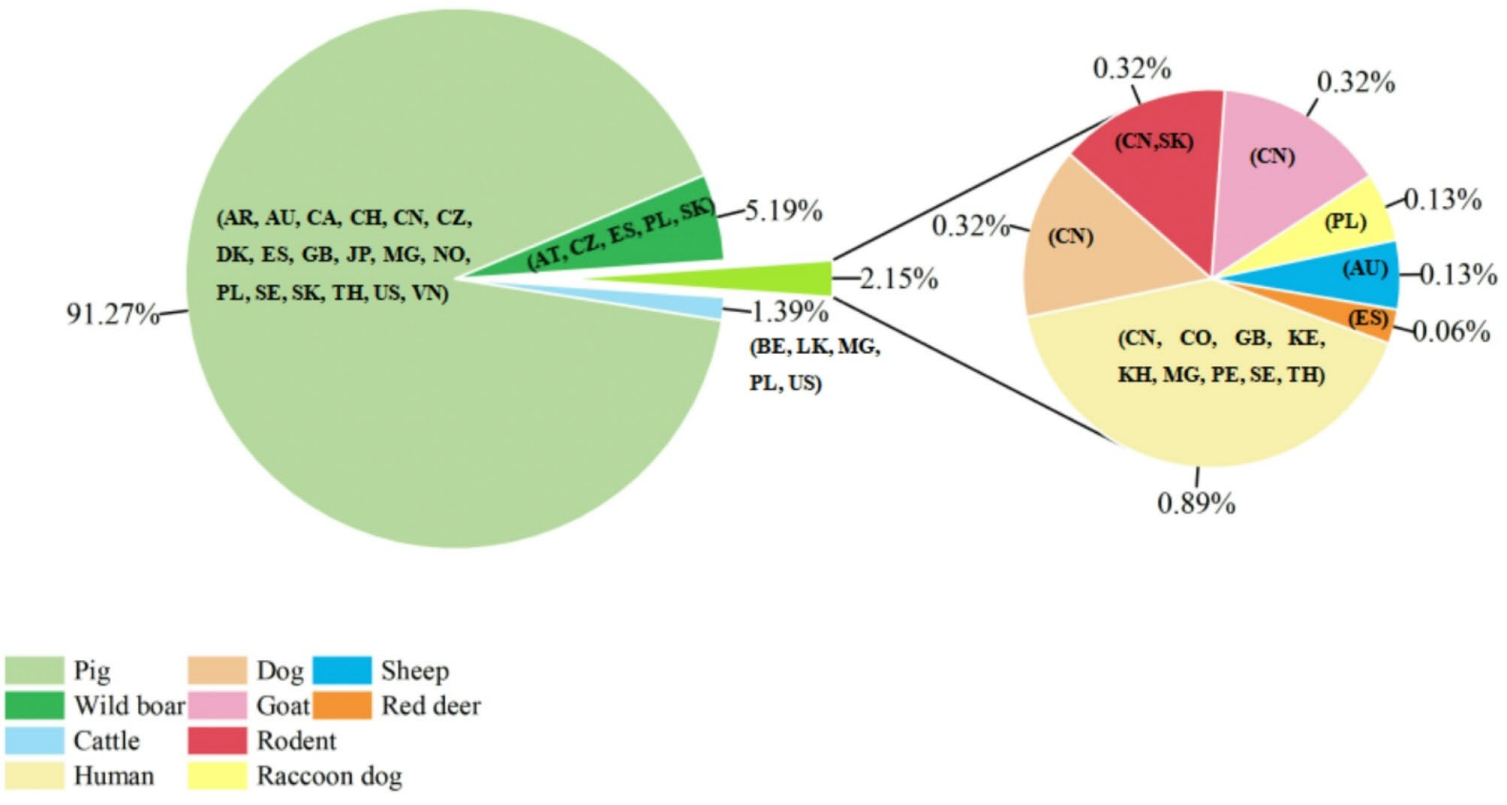
Pie charts showing the percentage of records per country and host species for the documented *C. suis*. The country codes (ISO 3166–1) stand for Argentina (AR), Austria (AT), Australia (AU), Belgium (BE), Canada (CA), Switzerland (CH), China (CN), Colombia (CO), Czech Republic (CZ), Denmark (DK), Spain (ES), the United Kingdom (GB), Japan (JP), Kenya (KE), Cambodia (KH), Sri Lanka (LK), Madagascar (MG), Norway (NO), Peru (PE), Poland (PL), Sweden (SE), Slovakia (SK), Thailand (TH), United States of America (US), Vietnam (VN)

The present study identified *C. suis* in yaks, Tibetan sheep, and horses in the investigated area. According to the published studies, to date, twelve *Cryptosporidium* species/genotypes (*C. bovis*, *Cryptosporidium ryanae*, *C. baileyi*, *C. andersoni*, *C. parvum*, *C. hominis*, *C. canis*, *Cryptosporidium struthionis*, *C. xiaoi*, *C. ubiquitum*, *C. suis-like*, and *Cryptosporidium* new genotype) have been identified in yaks [20]. Fourteen *Cryptosporidium* species/genotypes (*C. parvum*, *C. ubiquitum*, *C. xiaoi*, *C. bovis*, *C. scrofarum*, *C. andersoni*, *C. hominis*, *C. canis*, *C. ryanae*, *C. suis*, *C. fayeri*, *C. meleagridis*, *C. muris*, and sheep genotype I) have been reported in sheep globally [77]. In addition, twelve *Cryptosporidium* species and two genotypes (*C. bovis*, *C. erinacei*, *C. felis*, *C. hominis*, *C. muris*, *C. parvum*, *C. andersoni*, *Cryptosporidium proliferans*, *C. ryanae*, *C. tyzzeri*, *C. ubiquitum*, *C. xiaoi*, *Cryptosporidium* pig genotype, and *Cryptosporidium* horse genotype) have been reported in horses [78, 79]. Surprisingly, this is the first report of *C. suis* infection in yaks and horses, and *C. suis* was the only species found in this study. The present study indicates that *C. suis* has a wider host range than initially believed and possesses the capacity to infect humans. To the best of our knowledge, pigs are the major hosts of *C. suis*, followed by wild boars. Furthermore, *C. suis* was also found in other domestic animals (cattle, dogs, goats, and sheep) and wildlife (rodents, raccoon dogs, and red deer) from 25 countries (Fig. 3, Additional file 2: Table S2). A published study showed that *C. suis* was detected in sheep in Australia [80]. Thus, we hypothesize that there was cross-species transmission of *C. suis* among yaks, Tibetan sheep, and horses, implying they may be reservoirs of human cryptosporidiosis.

In this study, three ITS genotypes (one known genotype and two novel genotypes) were identified in yaks and Tibetan sheep (only BEB4) in Shiqu, with BEB4 being the predominant genotype (identified in 8 of the 11 positive samples), which is consistent with that reported in yaks in Qinghai, China [81], and cattle in the USA [82]. Of these genotypes, genotype SQY1 belonged to human-pathogenic Group 1, which implied that the yaks may be a potential source of human infection. Although the remaining genotypes (BEB4 and SQY2) were classified into Group 2, we must be cautious because the cattle-specific genotype (BEB4) has been identified in humans [83, 84], and it has also been found in water buffalo [16], nonhuman primates [85], pigs [83], donkeys [86], and raccoon dogs [87]. However, interestingly genotype BEB4 was firstly detected in Tibetan sheep [5]. Genotype BEB6, to our knowledge, has been most commonly reported in sheep, but the results in our study were in contrast to the published studies [5, 88], which suggested that there was cross-species transmission and a potential threat to humans. On the one hand, all genotypes identified in this study have a possible zoonotic potential, implying that these animals play an important role as reservoir hosts in *E. bieneusi* transmission to humans; on the other hand, after yaks, Tibetan sheep, and horses were infected with *E. bieneusi*, their health was affected, presenting heavy economic losses and impact to animal production and local herdsmen. Nevertheless, more epidemiologic studies are needed to understand the source and transmission dynamics of *E. bieneusi* and assess the role of livestock in the transmission of human microsporidiosis.

## Conclusions

The present study investigated the prevalence and performed molecular characterization of *Cryptosporidium* spp. and *E. bieneusi* in yaks, Tibetan sheep, and horses from Shiqu. This study represents the first report of *C. suis* infection in yaks and horses, while also documenting the identification of *E. bieneusi* genotype BEB4 in Tibetan sheep worldwide. These findings demonstrate that the host ranges of these two protozoa are wider than previously reported. The present identification of zoonotic *C. suis* and *E. bieneusi* genotype BEB4 in this study suggests that these infected animals may be significant sources of infection of human cryptosporidiosis caused by *C. suis* and human microsporidiosis caused by *E. bieneusi* genotype BEB4 and may pose a threat to public health. More areas and a larger number of samples are required to assess the potential risk of cross-species transmission in the investigated areas in the future.

## Electronic supplementary material

Below is the link to the electronic supplementary material.

Supplementary Material 1

Supplementary Material 2

## Data Availability

The representative nucleotide sequences obtained in the present study were deposited in GenBank database under the following accession nos.: PQ637271 - PQ637286 for *C. suis*, and PQ628064 - PQ628074 for *E. bieneusi*.

## Abbreviations

PCR: Polymerase chain reaction
SSU rRNA: Small subunit ribosomal RNA
ITS: Internal transcribed spacer
