# Supplementary material for: Investigation of Cryptosporidium spp. and Enterocytozoon bieneusi in free-ranged livestock on the southeastern Qinghai–Xizang Plateau, China
Source: BMC Infect Dis. 2025 Mar 13;25:356. doi: 10.1186/s12879-025-10737-5 (PMC11907973; doi:10.1186/s12879-025-10737-5)
Supplement: Supplementary file 1 — Supplementary Material 1 [file 12879_2025_10737_MOESM1_ESM.docx]

Table S1 PCR primers used in this study

| **Gene locus** | **Primer** | **Sequence (5′–3′)** | **Amplicon length (bp)** | **Annealing temperature (◦C)** | **Reference** |
| --- | --- | --- | --- | --- | --- |
| SSU rRNA | F1 | TTCTAGAGCTAATACATGCGAA | 1325 | 55 | Huang et al., 2016 |
|  | R1 | CTCATAAGGTGCTGAAGG |  |  |  |
|  | F2 | GGAAGGGTTGTATTTATTAGATAAAG | ~830 | 55 |  |
|  | R2 | AGTAAGGAACAACCTCCC |  |  |  |
| ITS | EbGeno-F1 | TTCAGATGGTCATAGGGATG | 465 | 53 | Mirjalali et al., 2015 |
|  | EbGeno-R1 | ATTAGAGCATTCCGTGAGG |  |  |  |
|  | EbGeno-F2 | TCGGCTCTGAATATCTATGG | ~410 | 55 |  |
|  | EbGeno-R2 | ATTCTTTCGCGCTCGTC |  |  |  |
