# Supplementary material for: Investigation of Cryptosporidium spp. and Enterocytozoon bieneusi in free-ranged livestock on the southeastern Qinghai–Xizang Plateau, China
Source: BMC Infect Dis. 2025 Mar 13;25:356. doi: 10.1186/s12879-025-10737-5 (PMC11907973; doi:10.1186/s12879-025-10737-5)
Supplement: Supplementary file 2 — Supplementary Material 2 [file 12879_2025_10737_MOESM2_ESM.docx]

Table S2 Host range and geographical distribution of *C. suis* worldwide

| Host | County distribution (no.) | Positive no. | Percentage % (95% CI) | Reference |
| --- | --- | --- | --- | --- |
| Human | Thailand (6), China (1), Peru (1), England (1), Madagascar (1), Cambodia (1), Sweden (1), Kenya (1), Colombia (1) | 14 | 0.89 (0.51~1.49) | [1-9] |
| Domestic Animals |  |  |  |  |
| Pigs | Madagascar (3), China (339), Czech Republic (833), Japan (21), Slovakia Republic (4), Australia (28), UK (30), Canada (43), Denmark (68), Switzerland (4), Argentina (7), Vietnam (2), Spain (20), Poland (7), USA (1), Sweden (17), Thailand (9), Norway (6) | 1442 | 91.27 (89.74~92.56) | [5, 10-47] |
| Cattle | Madagascar (17), the United States (1), Sri Lanka (1), Poland (2), Belgium (1) | 22 | 1.39 (0.89~2.13) | [5, 48-51] |
| Dogs | China (5) | 5 | 0.32 (0.12~0.74) | [52] |
| Goats | China (5) | 5 | 0.32 (0.12~0.74) | [53] |
| Sheep | Australia (2) | 2 | 0.13 (0.02~0.47) | [54] |
| Wildlife |  |  |  |  |
| Wild boars | Czech Republic (25), Spain (20), Europe*^a^* (35), Slovakia Republic (2) | 82 | 5.19 (4.17~6.43) | [55-60] |
| Rodents | Slovakia  Republic (4), China (1) | 5 | 0.32(0.12~0.74) | [61, 62] |
| Raccoon dogs | Poland (2) | 2 | 0.13 (0.02~0.47) | [63] |
| Red deer | Spain (1) | 1 | 0.06 (0.01~0.35) | [64] |
| Total |  | 1580 | 100.00 |  |

*^a^* including Austria, the Czech Republic, Poland, and the Slovak Republic

**Reference**

1. Sannella AR, Suputtamongkol Y, Wongsawat E, Cacciò SM. A retrospective molecular study of *Cryptosporidium* species and genotypes in HIV-infected patients from Thailand. Parasite Vectors. 2019;12(1): 91. https://doi.org/10.1186/s13071-019-3348-4.

2. Wang L, Zhang H, Zhao X, Zhang L, Zhang G, Guo M, et al. Zoonotic *Cryptosporidium* species and *Enterocytozoon bieneusi* genotypes in HIV-positive patients on antiretroviral therapy. J Clin Microbiol. 2013;51(2): 557–563. https://doi.org/10.1128/JCM.02758-12.

3. Cama VA, Ross JM, Crawford S, Kawai V, Chavez-Valdez R, Vargas D, et al. Differences in clinical manifestations among *Cryptosporidium* species and subtypes in HIV-infected persons. J Infect Dis. 2007;196(5). https://doi.org/10.1086/519842.

4. Leoni F, Amar C, Nichols G, Pedraza-Díaz S, McLauchlin J. Genetic analysis of *Cryptosporidium* from 2414 humans with diarrhoea in England between 1985 and 2000. J Med Microbiol. 2006;55(6): 703–707. https://doi.org/10.1099/jmm.0.46251-0.

5. Bodager JR, Parsons MB, Wright PC, Rasambainarivo F, Roellig D, Xiao L, et al. Complex epidemiology and zoonotic potential for *Cryptosporidium* suis in rural Madagascar. Vet parasitol. 2015;207(1–2). https://doi.org/10.1016/j.vetpar.2014.11.013.

6. Moore CE, Elwin K, Phot N, Seng C, Mao S, Suy K, et al. Molecular characterization of *Cryptosporidium* species and *Giardia* *duodenalis* from symptomatic Cambodian Children. PLoS Negl Trop Dis. 2016;10(7). https://doi.org/10.1371/journal.pntd.0004822.

7. Lebbad M, Winiecka-Krusnell J, Stensvold CR, Beser J. High diversity of *Cryptosporidium* species and subtypes identified in Cryptosporidiosis acquired in Sweden and Abroad. Pathogens. 2021;10(5): 523. https://doi.org/10.3390/pathogens10050523.

8. Uran-Velasquez J, Alzate JF, Farfan-Garcia AE, Gomez-Duarte OG, Martinez-Rosado LL, Dominguez-Hernandez DD, et al. Multilocus sequence typing helps understand the genetic diversity of *cryptosporidium hominis* and *cryptosporidium* *parvum* isolated from colombian patients. PLoS One. 2022;17(7): e0270995. https://doi.org/10.1371/journal.pone.0270995.

9. Squire SA, Ryan U. *Cryptosporidium* and *Giardia* in Africa: current and future challenges. Parasite Vectors. 2017;10(1): 195. https://doi.org/10.1186/s13071-017-2111-y.

10. Lin Q, Wang XY, Chen JW, Ding L, Zhao GH. *Cryptosporidium* *suis i*nfection in post-weaned and adult pigs in shaanxi province, northwestern China. Korean J Parasitol. 2015;53(1): 113–117. https://doi.org/10.3347/kjp.2015.53.1.113.

11. Němejc K, Sak B, Květoňová D, Kernerová N, Rost M, Cama VA, et al. Occurrence of *Cryptosporidium* *suis* and *Cryptosporidium* *scrofarum* on commercial swine farms in the Czech Republic and its associations with age and husbandry practices. Parasitol Res. 2013;112(3): 1143–1154. https://doi.org/10.1007/s00436-012-3244-8.

12. Kvác M, Hanzlíková D, Sak B, Kvetonová D. Prevalence and age-related infection of *Cryptosporidium* *suis*, *C. muris* and *Cryptosporidium* pig genotype II in pigs on a farm complex in the Czech Republic. Vet parasitol. 2009;160(3–4). https://doi.org/10.1016/j.vetpar.2008.11.007.

13. Yui T, Nakajima T, Yamamoto N, Kon M, Abe N, Matsubayashi M, et al. Age-related detection and molecular characterization of *Cryptosporidium suis* and *Cryptosporidium* *scrofarum* in pre- and post-weaned piglets and adult pigs in Japan. Parasitol Res. 2014;113(1): 359–365. https://doi.org/10.1007/s00436-013-3662-2.

14. Danišová O, Valenčáková A, Petrincová A. Detection and identification of six Cryptospordium species in livestock in Slovakia by amplification of SSU and GP60 genes with the use of PCR analysis. Ann Agric Environ Med. 2016;23(2). https://doi.org/10.5604/12321966.1203886.

15. Ryan U, Xiao L, Read C, Zhou L, Lal AA, Pavlasek I. Identification of novel *Cryptosporidium* genotypes from the Czech Republic. Appl Environ Microbiol. 2003;69(7): 4302–4307. https://doi.org/10.1128/AEM.69.7.4302-4307.2003.

16. Xiao L, Moore JE, Ukoh U, Gatei W, Lowery CJ, Murphy TM, et al. Prevalence and identity of *Cryptosporidium* spp. in pig slurry. Appl Environ Microbiol. 2006;72(6): 4461–4463. https://doi.org/10.1128/AEM.00370-06.

17. Johnson J, Buddle R, Reid S, Armson A, Ryan UM. Prevalence of *Cryptosporidium* genotypes in pre and post-weaned pigs in Australia. Exp Parasitol. 2008;119(3): 418–421. https://doi.org/10.1016/j.exppara.2008.04.009.

18. Budu-Amoako E, Greenwood SJ, Dixon BR, Barkema HW, Hurnik D, Estey C, et al. Occurrence of *Giardia* and *Cryptosporidium* in pigs on Prince Edward Island, Canada. Vet Parasitol. 2012;184(1): 18–24. https://doi.org/10.1016/j.vetpar.2011.07.047.

19. Zhang W, Yang F, Liu A, Wang R, Zhang L, Shen Y, et al. Prevalence and genetic characterizations of *Cryptosporidium* spp. in pre-weaned and post-weaned piglets in Heilongjiang province, China. PloS One. 2013;8(7): e67564. https://doi.org/10.1371/journal.pone.0067564.

20. Petersen HH, Jianmin W, Katakam KK, Mejer H, Thamsborg SM, Dalsgaard A, et al. *Cryptosporidium* and *Giardia* in Danish organic pig farms: seasonal and age-related variation in prevalence, infection intensity and species/genotypes. Vet Parasitol. 2015;214(1–2): 29–39. https://doi.org/10.1016/j.vetpar.2015.09.020.

21. Schubnell F, von Ah S, Graage R, Sydler T, Sidler X, Hadorn D, et al. Occurrence, clinical involvement and zoonotic potential of endoparasites infecting Swiss pigs. Parasitol Int. 2016;65(6 Pt A): 618–624. https://doi.org/10.1016/j.parint.2016.09.005.

22. Qi M, Zhang Q, Xu C, Zhang Y, Xing J, Tao D, et al. Prevalence and molecular characterization of *Cryptosporidium* spp. in pigs in Xinjiang, China. Acta Tropica. 2020;209: 105551. https://doi.org/10.1016/j.actatropica.2020.105551.

23. De Felice LA, Moré G, Cappuccio J, Venturini MC, Unzaga JM. Molecular characterization of *Cryptosporidium* spp. from domestic pigs in Argentina. Vet Parasitol Reg Stud Reports. 2020;22: 100473. https://doi.org/10.1016/j.vprsr.2020.100473.

24. Iwashita H, Takemura T, Tokizawa A, Sugamoto T, Thiem VD, Nguyen TH, et al. Molecular epidemiology of *Cryptosporidium* spp. in an agricultural area of northern Vietnam: a community survey. Parasitol Int. 2021;83. https://doi.org/10.1016/j.parint.2021.102341.

25. Li D, Deng H, Zheng Y, Zhang H, Wang S, He L, et al. First characterization and zoonotic potential of *Cryptosporidium* spp. and *Giardia* *duodenalis* in pigs in Hubei province of China. Front Cell Infect Microbiol. 2022;12: 949773. https://doi.org/10.3389/fcimb.2022.949773.

26. Vítovec J, Hamadejová K, Landová L, Kvác M, Kvetonová D, Sak B. Prevalence and pathogenicity of *Cryptosporidium suis* in pre- and post-weaned pigs. J Vet Med B Infect Dis Vet Public Health. 2006;53(5): 239–243. https://doi.org/10.1111/j.1439-0450.2006.00950.x.

27. Suárez-Luengas L, Clavel A, Quílez J, Goñi-Cepero MP, Torres E, Sánchez-Acedo C, et al. Molecular characterization of *Cryptosporidium* isolates from pigs in Zaragoza (northeastern Spain). Vet Parasitol. 2007;148(3–4): 231–235. https://doi.org/10.1016/j.vetpar.2007.06.022.

28. Langkjaer RB, Vigre H, Enemark HL, Maddox-Hyttel C. Molecular and phylogenetic characterization of *Cryptosporidium* and *Giardia* from pigs and cattle in Denmark. Parasitology. 2007;134(Pt 3): 339–350. https://doi.org/10.1017/S0031182006001533.

29. Zintl A, Neville D, Maguire D, Fanning S, Mulcahy G, Smith HV, et al. Prevalence of *Cryptosporidium* species in intensively farmed pigs in Ireland. Parasitology. 2007;134(Pt 11): 1575–1582. https://doi.org/10.1017/S0031182007002983.

30. Kvác M, Sak B, Hanzlíková D, Kotilová J, Kvetonová D. Molecular characterization of *Cryptosporidium* isolates from pigs at slaughterhouses in South Bohemia, Czech Republic. Parasitol Res. 2009;104(2): 425–428. https://doi.org/10.1007/s00436-008-1215-x.

31. Wang R, Qiu S, Jian F, Zhang S, Shen Y, Zhang L, et al. Prevalence and molecular identification of *Cryptosporidium* spp. in pigs in Henan, China. Parasitol Res. 2010;107(6): 1489–1494. https://doi.org/10.1007/s00436-010-2024-6.

32. Farzan A, Parrington L, Coklin T, Cook A, Pintar K, Pollari F, et al. Detection and characterization of *Giardia* *duodenalis* and *Cryptosporidium* spp. on swine farms in Ontario, Canada. Foodborne Pathog Dis. 2011;8(11): 1207–1213. https://doi.org/10.1089/fpd.2011.0907.

33. Yin JH, Yuan ZY, Cai HX, Shen YJ, Jiang YY, Zhang J, et al. Age-related infection with *Cryptosporidium* species and genotype in pigs in China. Biomed Environ Sci. 2013;26(6): 492–495. https://doi.org/10.3967/0895-3988.2013.06.010.

34. Rzeżutka A, Kaupke A, Kozyra I, Pejsak Z. Molecular studies on pig cryptosporidiosis in Poland. Pol J Vet Sci. 2014;17(4): 577–582. https://doi.org/10.2478/pjvs-2014-0086.

35. Rodriguez-Rivera LD, Cummings KJ, McNeely I, Suchodolski JS, Scorza AV, Lappin MR, et al. Prevalence and diversity of *Cryptosporidium* and *Giardia* identified among feral pigs in Texas. Vector Borne Zoonotic Dis. 2016;16(12): 765–768. https://doi.org/10.1089/vbz.2016.2015.

36. Wang H, Zhang Y, Wu Y, Li J, Qi M, Li T, et al. Occurrence, molecular mharacterization, and assessment of zoonotic risk of *Cryptosporidium* spp., *Giardia duodenalis*, and *Enterocytozoon bieneusi* in Pigs in Henan, Central China. J Eukaryot Microbiol. 2018;65(6): 893–901. https://doi.org/10.1111/jeu.12634.

37. Zheng S, Li D, Zhou C, Zhang S, Wu Y, Chang Y, et al. Molecular identification and epidemiological comparison of *Cryptosporidium* spp. among different pig breeds in Tibet and Henan, China. BMC Vet Res. 2019;15(1): 101. https://doi.org/10.1186/s12917-019-1847-3.

38. Pettersson E, Ahola H, Frössling J, Wallgren P, Troell K. Detection and molecular characterisation of *Cryptosporidium* spp. in Swedish pigs. Acta Vet Scand. 2020;62(1): 40. https://doi.org/10.1186/s13028-020-00537-z.

39. Thathaisong U, Siripattanapipong S, Inpankaew T, Leelayoova S, Mungthin M. High prevalence of *Cryptosporidium* infection caused by *C. scrofarum* and *C. suis* among pigs in Thailand. Parasitol Int. 2020;77: 102122. https://doi.org/10.1016/j.parint.2020.102122.

40. Wang P, Li S, Zou Y, Du ZC, Song DP, Wang P, et al. The infection and molecular characterization of *Cryptosporidium* spp. in diarrheic pigs in southern China. Microb Pathog. 2022;165: 105459. https://doi.org/10.1016/j.micpath.2022.105459.

41. Lam HYP, Tseng YC, Wu WJ, Yu YH, Cheng PC, Peng SY. Prevalence and genotypes of *Cryptosporidium* in livestock in Hualien Country, Eastern Taiwan. Parasitol Int. 2022;88: 102553. https://doi.org/10.1016/j.parint.2022.102553.

42. Zhang Y, Xu C, Xing J, Ao W, Qi M, Jing B. PCR Detection of *Cryptosporidium* spp.and *Giardia duodenalis* in A Xiang Pig Farm of Aksu. Progress in Veterinary Medicine. 2020;41(11):68-71.https://doi.org/10.16437/j.cnki.1007-5038.2020.11.013.

43. Yao Q, Wang J, Wang S, Li Y, Zhao S, Song J, et al. Analysis of *Cryptosporidium* infection in piglets from partial regions of Shaanxi Province. Chinese Journal of Veterinary Science. 2020;40(12):2348-2352.https://doi.org/10.16303/j.cnki.1005-4545.2020.12.13.

44. Ryan U, Read C, Hawkins P, Warnecke M, Swanson P, Griffith M, et al. Genotypes of *Cryptosporidium* from Sydney water catchment areas. J Appl Microbiol. 2005;98(5): 1221–1229. https://doi.org/10.1111/j.1365-2672.2005.02562.x.

45. Hamnes IS, Gjerde BK, Forberg T, Robertson LJ. Occurrence of *Cryptosporidium* and *Giardia* in suckling piglets in Norway. Vet Parasitol. 2007;144(3–4): 222–233. https://doi.org/10.1016/j.vetpar.2006.10.011.

46. Hao Y, Liu A, Li H, Zhao Y, Yao L, Yang B, et al. Molecular characterization and zoonotic potential of *Cryptosporidium* spp. and *Giardia duodenalis* in humans and domestic animals in Heilongjiang Province, China. Parasite Vectors. 2024;17(1): 155. https://doi.org/10.1186/s13071-024-06219-3.

47. Rivero-Juárez A, Dashti A, Santín M, Köster PC, López-López P, Risalde MA, et al. Diarrhoea-causing enteric protist species in intensively and extensively raised pigs (*Sus scrofa* domesticus) in Southern Spain. Part II: Association with Hepatitis E virus susceptibility. Transbound Emerg Dis. 2022;69(4): e1172–e1178. https://doi.org/10.1111/tbed.14408.

48. Fayer R, Santín M, Trout JM, Greiner E. Prevalence of species and genotypes of *Cryptosporidium* found in 1–2-year-old dairy cattle in the eastern United States. Vet Parasitol. 2006;135(2): 105–112. https://doi.org/10.1016/j.vetpar.2005.08.003.

49. Abeywardena H, Jex AR, Koehler AV, Rajapakse RJ, Udayawarna K, Haydon SR, et al. First molecular characterization of *Cryptosporidium* and *Giardia* from bovines (*Bos taurus* and *Bubalus bubalis*) in Sri Lanka: unexpected absence of *C. parvum* from pre-weaned calves. Parasite Vectors. 2014;7(1): 75. https://doi.org/10.1186/1756-3305-7-75.

50. Rzeżutka A, Kaupke A. *Cryptosporidium* infections in asymptomatic calves up to 4 months in Poland: a cross-sectional population study. Sci Rep. 2023;13(1): 20997. https://doi.org/10.1038/s41598-023-47810-5.

51. Geurden T, Berkvens D, Martens C, Casaert S, Vercruysse J, Claerebout E. Molecular epidemiology with subtype analysis of *Cryptosporidium* in calves in Belgium. Parasitology. 2007 Dec;134(Pt.14):1981-7. https://doi.org/10.1017/S0031182007003460.

52. Jian J, Liu A, Yang Y, Peng X, Yao L, Li B, et al. Occurrence rate and species and subtypes of *Cryptosporidium* spp. in pet dogs in yunnan province, China. BMC Microbiol. 2024;24(1): 354. https://doi.org/10.1186/s12866-024-03500-4.

53. Zhong Z, Tu R, Ou H, Yan G, Dan J, Xiao Q, et al. Occurrence and genetic characterization of *Giardia duodenalis* and *Cryptosporidium* spp. from adult goats in Sichuan Province, China. PLoS One. 2018;13(6): e0199325. https://doi.org/10.1371/journal.pone.0199325.

54. Ryan UM, Bath C, Robertson I, Read C, Elliot A, Mcinnes L, et al. Sheep may not be an important zoonotic reservoir for *Cryptosporidium* and *Giardia* parasites. Appl Environ Microbiol. 2005;71(9): 4992–4997. https://doi.org/10.1128/AEM.71.9.4992-4997.2005.

55. Němejc K, Sak B, Květoňová D, Hanzal V, Jeníková M, Kváč M. The first report on *Cryptosporidium* *suis* and *Cryptosporidium* pig genotype II in Eurasian wild boars (*Sus scrofa*) (Czech Republic). Vet Parasitol. 2012;184(2–4): 122–125. https://doi.org/10.1016/j.vetpar.2011.08.029.

56. García-Presedo I, Pedraza-Díaz S, González-Warleta M, Mezo M, Gómez-Bautista M, Ortega-Mora LM, et al. Presence of *Cryptosporidium* *scrofarum*, *C. suis* and *C. parvum* subtypes IIaA16G2R1 and IIaA13G1R1 in Eurasian wild boars (*Sus scrofa*). Vet Parasitol. 2013;196(3–4): 497–502. https://doi.org/10.1016/j.vetpar.2013.04.017.

57. Němejc K, Sak B, Květoňová D, Hanzal V, Janiszewski P, Forejtek P, et al. *Cryptosporidium* *suis* and *Cryptosporidium scrofarum* in Eurasian wild boars (*Sus scrofa*) in Central Europe. Vet Parasitol. 2013;197(3–4): 504–508. https://doi.org/10.1016/j.vetpar.2013.07.003.

58. Rivero-Juarez A, Dashti A, López-López P, Muadica AS, Risalde M de LA, Köster PC, et al. Protist enteroparasites in wild boar (*Sus scrofa ferus*) and black Iberian pig (*Sus scrofa* domesticus) in southern Spain: a protective effect on hepatitis E acquisition? Parasite Vectors. 2020;13(1): 281. https://doi.org/10.1186/s13071-020-04152-9.

59. Martí-Marco A, Moratal S, Torres-Blas I, Cardells J, Lizana V, Dea-Ayuela MA. Molecular detection and epidemiology of potentially zoonotic *Cryptosporidium* spp. and *Giardia duodenalis* in wild boar (*Sus scrofa*) from eastern spain. Animals. 2023;13(15): 2501. https://doi.org/10.3390/ani13152501.

60. Valenčáková A, Sučik M, Danišová O, Kandráčová P, Tomko M, Valocký I. Detection of *Blastocystis* spp., *Cryptosporidium* spp. and *Encephalitozoon* spp. among wild animals from eastern Slovakia. Acta Vet Hung. 2022;70(3): 220–225. https://doi.org/10.1556/004.2022.00026.

61. Danišová O, Valenčáková A, Stanko M, Luptáková L, Hatalová E, Čanády A. Rodents as a reservoir of infection caused by multiple zoonotic species/genotypes of *C. parvum*, *C. hominis*, *C. suis*, *C. scrofarum*, and the first evidence of *C.* muskrat genotypes I and II of rodents in Europe. Acta Trop. 2017;172: 29–35. https://doi.org/10.1016/j.actatropica.2017.04.013.

62. Feng S, Chang H, Wang Y, Huang C, Han S, He H. Molecular characterization of *Cryptosporidium* spp. in brandt’s vole in China. Front Vet Sci. 2020;7: 300.

https://doi.org/10.3389/fvets.2020.00300.

63. Perec-Matysiak A, Hildebrand J, Popiołek M, Buńkowska-Gawlik K. The occurrence of *Cryptosporidium* spp. in wild-living carnivores in poland—a question concerning its host specificity. Pathogens. 2023;12(2): 198. https://doi.org/10.3390/pathogens12020198.

64. Dashti A, Köster PC, Bailo B, de Las Matas AS, Habela MÁ, Rivero-Juarez A, et al. Occurrence and limited zoonotic potential of *Cryptosporidium* spp., *Giardia duodenalis*, and *Balantioides coli* infections in free-ranging and farmed wild ungulates in Spain. Res Vet Sci. 2023;159: 189–197. https://doi.org/10.1016/j.rvsc.2023.04.020.
